# FBG-Based Temperature Sensors for Liquid Identification and Liquid Level Estimation via Random Forest

**DOI:** 10.3390/s21134568

**Published:** 2021-07-03

**Authors:** Katiuski Pereira, Wagner Coimbra, Renan Lazaro, Anselmo Frizera-Neto, Carlos Marques, Arnaldo Gomes Leal-Junior

**Affiliations:** 1 Graduate Program in Electrical Engineering, Federal University of Espirito Santo (UFES), Vitória 29075-910, ES, Brazil; katiuski.p@hotmail.com (K.P.); wagner.moraes@edu.ufes.br (W.C.); renan.lazaro@aluno.ufes.br (R.L.); frizera@ieee.org (A.F.-N.); 2 Physics Department & I3N, University of Aveiro, 3810-193 Aveiro, Portugal; carlos.marques@ua.pt

**Keywords:** fiber Bragg gratings, temperature sensor, random forest, oil classification, fluid identification, liquid level estimation

## Abstract

This paper proposed a liquid level measurement and classification system based on a fiber Bragg grating (FBG) temperature sensor array. For the oil classification, the fluids were dichotomized into oil and nonoil, i.e., water and emulsion. Due to the low variability of the classes, the random forest (RF) algorithm was chosen for the classification. Three different fluids, namely water, mineral oil, and silicone oil (Kryo 51), were identified by three FBGs located at 21.5 cm, 10.5 cm, and 3 cm from the bottom. The fluids were heated by a Peltier device placed at the bottom of the beaker and maintained at a temperature of 318.15 K during the entire experiment. The fluid identification by the RF algorithm achieved an accuracy of 100%. An average root mean squared error (RMSE) of 0.2603 cm, with a maximum RMSE lower than 0.4 cm, was obtained in the fluid level measurement also using the RF algorithm. Thus, the proposed method is a feasible tool for fluid identification and level estimation under temperature variation conditions and provides important benefits in practical applications due to its easy assembly and straightforward operation.

## 1. Introduction

Liquid level sensing is an important task in many industries, such as agriculture, automobile, food, storage, chemical, medical, oil, and gas [1]. An accurate level measurement can be critical to prevent environmental hazards, increase operational efficiency, and enhance performance [2]. Liquid level sensors are responsible for detecting the interface level in vessels, tanks, wells, reservoirs, and hoppers [3], e.g., in the oil and gas industry, in which water, oils, and gases, with different properties, such as density and viscosity, are processed in the same tank [3]. Crude oil processing produces flammable gases with explosion risk, requiring more complex and more robust sensors for interface level measuring [4].

Different methods for measuring the liquid level, based on acoustic, infrared, X-ray, ultrasonic, mechanical, electrical, capacitive, and optical technologies, have been reported [2,5,6,7,8,9]. Electrical liquid level sensors have been employed in most applications due to their high commercial availability and low cost. Electronic sensors suffer nonetheless from intrinsic safety concerns in the harsh environments of the oil and gas industry, especially with corrosive or flammable atmospheres [4]. For that reason, capacitive and optical sensors are frequently proposed to measure the interface level in oil tanks, since neither technique uses electric current to perform the measurement [1,9].

Fiber optic-based liquid level sensors (FOLLSs) provide advantageous characteristics demanded by the oil and gas industry, such as intrinsic safety, chemical corrosion resistance, electromagnetic interference immunity, electrical insulation, small size, and easy multiplexing capacity and remote monitoring capabilities [4]. Different approaches and operation principles, such as Fabry–Perot interferometry (FPI) [10,11], Mach–Zehnder interferometry (MZI) [12], and fiber Bragg gratings (FBGs), are used in fiber optic sensors to measure numerous parameters, e.g., temperature [13], pressure [14], vibration [15], strains [16], density [11], thermal conductivity [17], and liquid level [1].

Regarding the FOLLSs, Díaz et. al. [4] reported an FBG-embedded diaphragm structure to estimate the liquid level in an industrial water tank. The operating principle of the sensor was based on the relation between the hydrostatic pressure generated by the liquids and the Bragg wavelength of the FBG sensors to estimate the water level in the tank. A similar FOLLS based on an FPI sensor was proposed in [18], in which an all-fused silica structure was composed of a lead-in single-mode fiber SMF, a silica ferrule, and a thin silica diaphragm used for large-range measurements. Despite the large-range liquid level sensing in harsh environments, the method is complex and expensive, since three components are fused with CO${}_{2}$ laser heating fusion bonding technology. A simpler methodology was reported in [19], where Archimedes’ law of buoyancy was applied to an FBG-based sensor, in which a reduction of the total load exerted by the suspended mass resulted in a strain variation in the FBG sensor. The three most promising optical technologies for liquid level monitoring, i.e., FPI, MZI, and FBG, were discussed in [4].

The FOLLSs, especially the ones based on FBGs, need a transduction mechanism in order to obtain the liquid level assessment, which can be achieved using cantilevers, floating devices, or diaphragms [4], where the latter is a popular mechanism for pressure and liquid level sensing [20]. Such designs lead to additional fabrication methods to incorporate the optical fiber sensors into the transduction structure. In the case of diaphragm-based sensors, there are additional parameters to be analyze, since the diaphragm fabrication and optical fiber incorporation can affect the sensor response [20]. Furthermore, the diaphragm material’s thermal and mechanical properties directly influence the sensor response [21]. It is also worth noting that in many FOLLS approaches, the liquid level estimation is obtained from the hydrostatic pressure, which is proportional to the fluid’s density [1]. Thus, for multiphase (or multifluid) liquids’ level assessment, multiple sensors (distributed along the tank) are needed [1], which further increases the system’s fabrication complexity. In general, optical fiber sensors also present temperature sensitivity. Therefore, FOLLSs generally need a temperature compensation technique to mitigate temperature cross-sensitivity in liquid level assessment [22]. For this reason, the direct estimation of the liquid level using only temperature sensors brings operational advantages (as additional fabrication and assemblies in the FBG are not necessary) and also leads to a higher spectral efficiency, as the same sensor is used for the temperature estimation, where the liquid level is estimated from the temperature data. In this case, the sensor is based on the differences between the temperature distribution and the thermal properties of liquids and air. To that extent, it is also possible to classify and estimate different fluids based on the thermal gradient differences between oil and water, for example [23].

The use of machine learning algorithms in FBG sensor applications has been widely employed. An extreme learning machine was applied in the separation of overlapping spectra generated at wavelength division multiplexing [24]. A Gaussian process regression was applied in temperature measurements with an FBG temperature sensor, obtaining improvements in the measurement accuracy and speed [25]. A support vector machine, along with particle swarm optimization, was applied to identify and locate pipeline leakage accidents using FBG hoop strain sensors [26].

Random forest (RF) is an ensemble learning algorithm, which is more robust and more accurate than algorithms that use unique learning [27]. In RF, multiple decision trees are combined to perform a regression from the average output of the trees or a classification by considering the votes of the trees. Since RF is based on decision trees, the RF algorithm can adapt to the nonlinear relations in the data to generate predictions [28]. Each decision tree is built using a bootstrap sample set, i.e., randomly selected samples with replacement, with only a number of randomly selected variables being used to produce the potential splits [27]. The error is thus reduced by using a model with low bias and decreasing its variance by the random forest algorithm, which reduces the overfitting risk. The variance is decreased by the combination of decision trees, and the correlations between trees are reduced by selecting different random subsets when building them. The number of selected variables in node splitting should be tuned to balance the decreasing correlation between trees and the effects on the bias and variance from using fewer attributes in prediction [27].

Aiming at this background and the advantages of liquid level classification and estimation using temperature responses, this paper proposed the use of a temperature sensor array, based on FBGs, for liquid level estimation and fluids’ classification. In this case, three different fluids (water, mineral, and silicone oil) were used, where one fluid was tested at a time. Machine learning algorithms were used to perform both the classification of the tested liquid and the regression of the liquid level inside the container. In this case, the RF algorithm was used due to its simplicity, as it can be implemented in local devices, without long training times. The FBG-based temperature sensors were characterized and applied to a tank with different fluids, where the fluids were classified (as oil or nonoil), then the level of each fluid was estimated.

## 2. Materials and Methods

**Figure 1:**
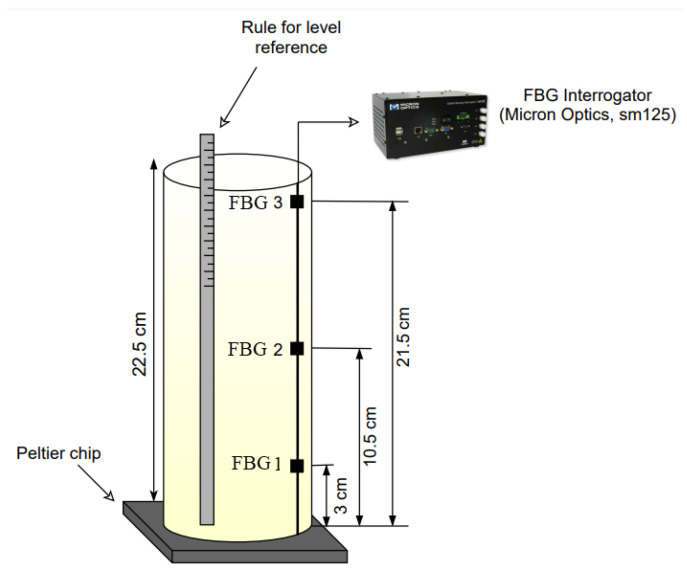
Experimental setup employed for fluid classification and level measurement.

The experimental analyses were performed using a beaker with a 2.2 cm radius and a 22.5 cm height filled with only one liquid. Water, mineral oil, and silicone oil Kryo 51 (LAUDA, Berlin, Germany) were the the fluids used for liquid level estimation in varying level conditions. Initially, each fluid was heated with a 12 W heat power provided by a Peltier heat sink TEC1-12706 (Hebei I.T., Shanghai, China), placed at the bottom of the beaker (see Figure 1), until it reached a temperature of 318.15 K, in order to simulate the heat flux variation common in crude oil tanks. As depicted in Figure 1, three FBGs were immersed in the fluids, located at 21.5 cm, 10.5 cm, and 3 cm from the bottom. In addition, an external FBG was used to measure the room temperature. An optical interrogator sm125 (Micron Optics, Atlanta, GA, USA), with a sampling rate of 2 Hz, was used to read the Bragg wavelength of the FBG sensors through the experiments. In this case, the Peltier plate was positioned beneath the container to emulate a temperature variation condition inside the container, which resembled the operation conditions in practical applications, where liquids at different temperatures are added in processing or storage tanks. Thus, the Peltier plate was not a component of the sensor system, which was only comprised of the FBGs and, therefore, could be used in the classified areas.

To fabricate the FBGs, the phase mask technique described in [29] was employed. Briefly, a photosensitive single-mode fiber (ThorLabs GF1B) was inscribed using a nanosecond-pulsed Nd:YAG laser emitting at 266 nm (LOTIS TII LS-2137ULaser) with an 8 ns pulse time. In order to make the grating inscription, the acrylate protection of the fiber needed to be removed, at around 45 mm, which also improved the sensitivity of the sensor due to the higher thermal conductivity of the silica when compared with the acrylate protection, which influenced the heat transfer from the liquids to the optical fiber. It is worth mentioning that the silica optical fiber without the acrylate protection was prone to damage and breakage due to the brittle nature of silica. However, in this case, the optical fiber was not subjected to large stresses or pressures, which greatly reduced the risk of fiber damage during the tests. The physical length of the FBG was 10 mm. In order to obtain the central Bragg wavelength of the fabricated FBGs, the FBG spectra were analyzed with the optical interrogator sm125 (Micron Optics, Atlanta, GA, USA). The Bragg wavelength ($\lambda_{B}$) measured is given by [30]:

(1) $$\begin{array}{c} \lambda_{B}=2n_{eff}\Lambda , \end{array}$$

where $n_{eff}$ is the effective refractive index and Λ is the Bragg grating period. Both $n_{eff}$ and Λ are affected by the strain and temperature of the grating by means of the elongation and thermal expansion effects of the material, which implies that Bragg wavelength shift $\Delta \lambda_{B}$ can be calculated by [7]:

(2) $$\begin{array}{c} \Delta \lambda_{B}=\lambda_{0}\left[\left(1-P_{\epsilon }\right)\epsilon +\left(\alpha +\zeta \right)\Delta T\right], \end{array}$$

where $\lambda_{0}$ is the initial value of the Bragg wavelength, $P_{\epsilon }$ is the effective photoelastic constant, ϵ is the strain applied to the gratings, α is the thermal expansion coefficient of the fiber material, ζ is the thermo-optic coefficient, and ΔT is the temperature variation. Since no stress was applied to the FBGs during all the experiments, strain measurements of the FBGs could be neglected, and Equation (2) can be rewritten as:

(3) $$\lambda_{B}=\lambda_{0}\left[1+\left(\alpha +\zeta \right)\Delta T\right].$$

The temperature characterization was performed with temperatures ranging from 298.15 K to 323.15 K in 5 K steps. Three measurement cycles consisting of increasing and decreasing temperatures were conducted in an immersion thermostat ECO RE 630 S (LAUDA, Berlin, Germany), without any forces acting on the fiber through the characterization. Subsequently, the sensor response to temperature, along with its repeatability, was analyzed by means of the wavelength shift caused by the temperature variations.

In the varying liquid level experiments, a measurement cycle consisted of first decreasing the liquid level from 22.5 cm to 0.9 cm from the bottom in steps of 5.4 cm, then increasing the level from 0.9 cm to 22.5 cm in the same steps of 5.4 cm. A total of three cycles were performed for each of the three liquids.

RF algorithms were used for both the classification of the fluids and the estimation of their levels. First, the fluid was identified, and then, the liquid level was estimated. For the classification, the RF algorithm was performed as discussed in our previous work [23]. The final prediction, oil or nonoil in this case, was decided by selecting the class that most of the trees identified as correct. For the regression, the final predicted level was calculated as the arithmetic mean of the tree forest results [31]. The physical principle of the fluid classification and liquid level estimation was based on the differences in the thermal properties of each fluid. These properties included the specific heat capacity and, especially, the thermal conductivity of the liquids. In a transient analysis, the liquids with different thermal properties resulted in differences in the temporal evolution of the wavelength shift. It was demonstrated in [17] that the thermal properties of the liquids can be estimated in different experimental conditions. For this reason, machine learning algorithms are able to detect such differences in the wavelength shift and classify the liquid in a straightforward approach. As it was based on the intrinsic properties of the fluids, the method can be applied to different containers and setup conditions.

For both the classification and regression by the random forest algorithm, the data samples were split into training data and testing data. The random forest algorithms were trained, i.e., the models were built using the data from the first two cycles and tested with the data from the last cycle. The data were obtained from the wavelength shifts of the corresponding fluids and liquid level measurements.

The RF construction for each tree is given by:

(4) $$\begin{array}{c} h(x,\theta_{k}),k=1,2,\ldots \end{array}$$

where *x* is the input vector and $\theta_{k}$ are the independent and identically distributed random vectors [27]. When RF increases the nodes of the classification tree, a better division of a random subset of input observations or predictive variables is chosen for the division of its nodes [32].

The RF classification uses the Gini index as the attribute selection measure. This index measures the degree of randomness of an attribute within a partition [27]. For an arbitrary set of T formations, randomly selected and belonging to class $C_{i}$, the Gini index can be written as:

(5) $$\begin{array}{c} \sum \sum_{j\neq i}=\left(\frac{f(C_{i},T)}{\left|T\right|}\right)\left(\frac{f(C_{j},T)}{\left|T\right|}\right) \end{array}$$

where f(Ci,T)/|T| is the probability that the selected case belongs to class $C_{i}$. To classify a new dataset, each new observation passes through each of the previously created *N* trees. The forest chooses the class with the highest number of votes and classifies it according to the vote.

The RF regression algorithm was used to estimate the level. A model was created for both the decreasing and increasing level of each liquid, and the liquid was selected by the fluid classification. In the model building, two different model approaches were used, namely Models 1 and 2. Model 1 considered all the FBG interactions, and Model 2 considered no interaction between the sensors. The variables present in Model 2 were each FBG, i.e., FBG 1, FBG 2, and FBG 3, whereas the ones of Model 2 were, besides the FBGs alone, (FBG 1) × (FBG 2), (FBG 1) × (FBG 3), (FBG 2) × (FBG 3), and (FBG 1) × (FBG 2) × (FBG 3).

In order to reduce the computational effort in the choice of the model, Model 2 was prioritized, because of its parsimony due to its fewer calculated coefficients, when the increase in RMSE of Model 2 in regard to Model 1 was lower than 15%.

## 3. Results and Discussion

**Figure 2:**
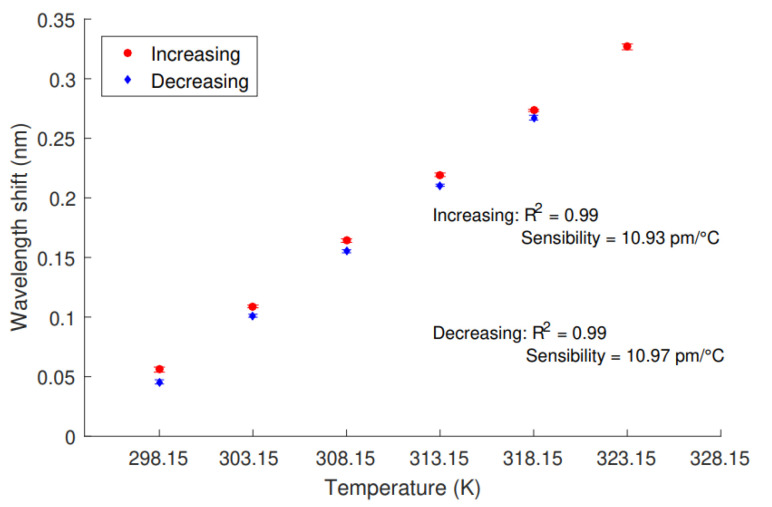
Increasing and decreasing temperature responses of the FBGs in the temperature characterization tests.

The temperature characterization of the FBG-based sensor is shown in Figure 2. The sensitivity calculated was 10.93 pm/ºC, with $R^{2}=0.99$ for both, increasing and decreasing. In addition, both coefficients of determination $R^{2}$ were greater than 99% for the linear regressions.

**Figure 3:**
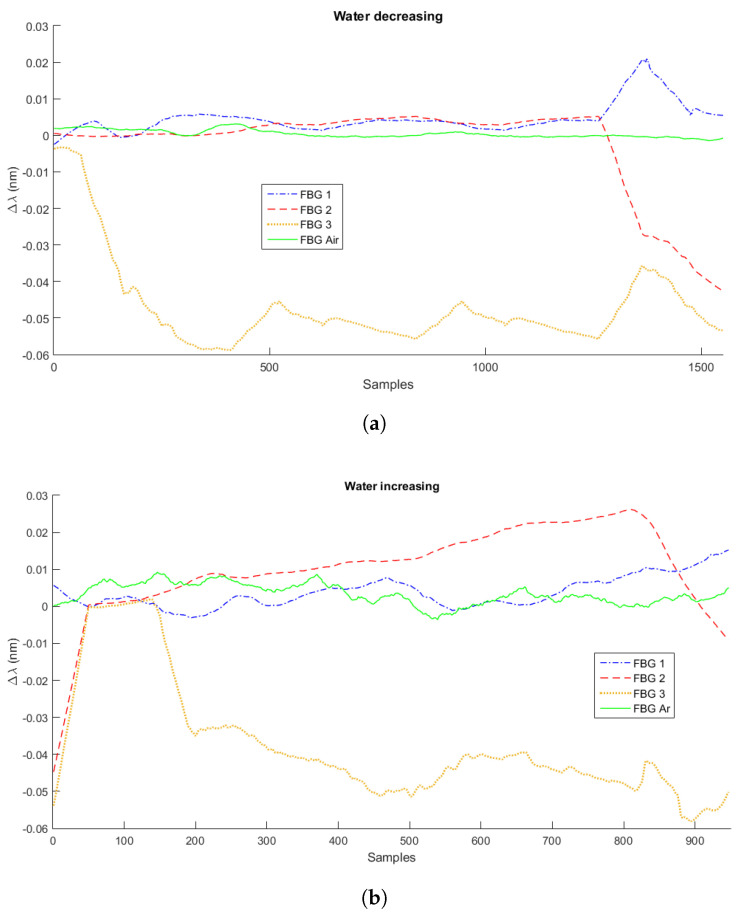
Measurements of decreasing (**a**) and increasing (**b**) liquid levels for water.

**Figure 4:**
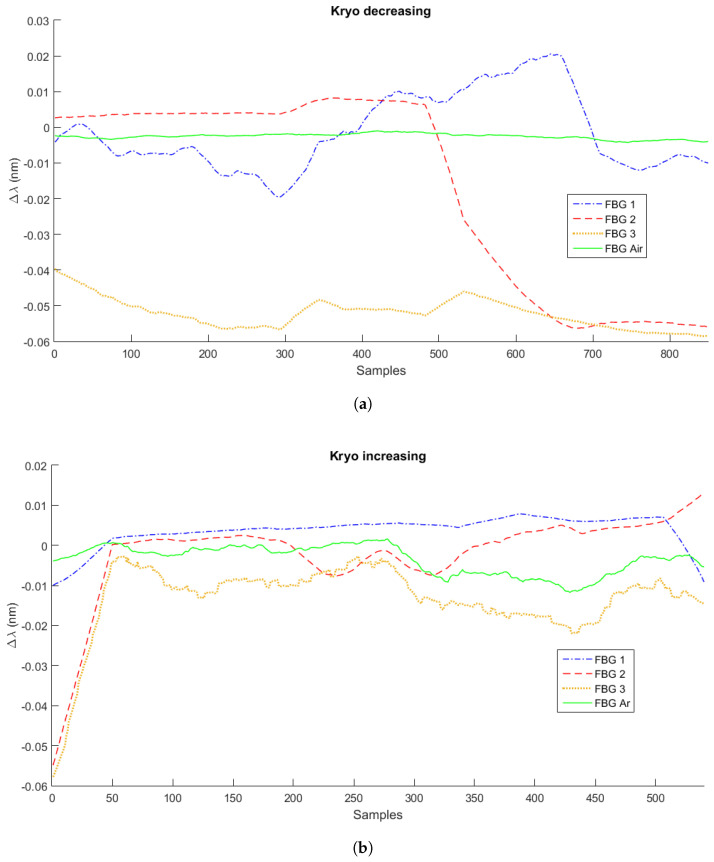
Measurements of decreasing (**a**) and increasing (**b**) liquid levels for Kryo 51.

**Figure 5:**
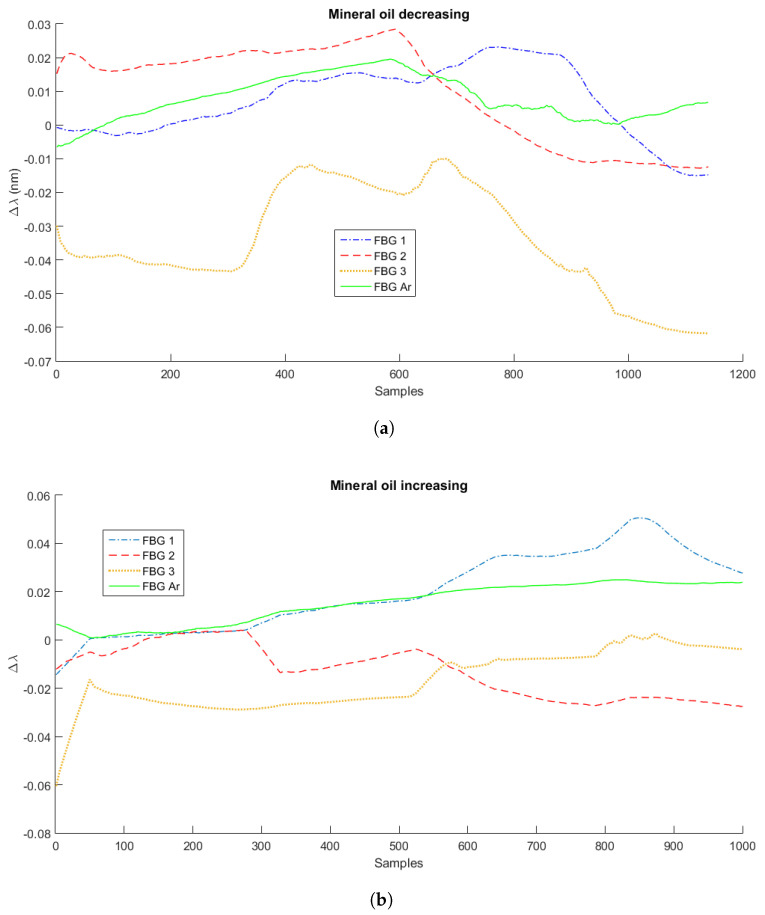
Measurements of decreasing (**a**) and increasing (**b**) liquid levels for mineral oil.

As mentioned in Section 2, the experiments were performed in three measurement cycles with three different liquids: water, mineral oil, and Kryo 51. Figure 3, Figure 4 and Figure 5 present the variation of the Bragg wavelength along the sampling time of the four FBGs used in the setup for the first measurement cycle, with a sampling rate of 2 Hz. Figure 3a,b presents the measurements of Cycle 1 of water’s decreasing and increasing level measurements, respectively. Figure 4a,b corresponds to the first Kryo 51 measurement cycle, and Figure 5a,b corresponds to that of mineral oil. The highest observable signal variation was when there was a variation of the surrounding fluid at each FBG, i.e., there was a higher wavelength shift when the FBG was dipped into the liquid or the liquid level reduced and the FBG was not submerged. However, these signal variations depended on the liquid heat transfer characteristics (such as the thermal conductivity) and depended on the heat transfer from the Peltier plate and surrounding environment. For these reasons, machine learning algorithms were used to increase the accuracy of the sensors’ response analyses.

The measurements of the first cycle were compared to those of the other two cycles by their corresponding standard errors (SEs). The obtained SEs between the three cycles were low, as expect for controlled experiments, with obtained standard errors of Cycles 2 and 3 lower than 0.2% with regard to Cycle 1.

The Pearson correlation coefficient ρ between the FBG measurements was calculated for the four FBG temperature sensors. The correlation coefficient represents the linear relations among the variables, and it is analyzed in order to avoid the multicollinearity of the data, which increases the variance of the model and the errors in the prediction. The correlation coefficients are shown in Table 1, Table 2 and Table 3 for the water, mineral oil, and Kryo 51, respectively. If the absolute value of ρ is greater than 0.85, one of the variables can be removed, in order to avoid duplicate information in the model [33].

The correlation coefficients between the FBG measurements were not greater than the limit of 0.85, except for the FBG–air in the oil measurements, the correlation coefficients of which were greater than 30% with all the other variables. Since FBG–air has no contact with the fluids, it was removed from the model building of the oil level. It can nevertheless be used as a reference sensor for the room temperature. In the water and Kryo 51 experiments, the calculated correlation of FBG 1 in regard to the other FBGs was greater than the correlations of the other FBGs. Since the correlations were less than 0.85, the variables were preserved, except for the FBG–air, which was used to monitor the room temperature. The liquid container had a heat input on the bottom (though the Peltier plate). However, the container walls were not thermally isolated (as also occurs in practical applications), which can lead to heat exchange with the environment. As the heat transfer occurred from the hot object to the colder one, there was a heat transfer from the container to the environment due to the higher temperature of the liquids inside the container (heated by the Peltier plate). Nevertheless, if there was a high increase of the room temperature in such a way that the room temperature was hotter than the liquids, the heat exchange would occur in the opposite direction, i.e., from the environment to the liquids through the liquid container walls, which resulted in two heat inputs: on the bottom from the Peltier plate and on the container walls. These different thermal dynamics can result in variations in the temperature gradient along the container, which may lead to errors in the liquid classification and level estimation. The assessment of room temperature was beneficial, since high variations in the room temperature can lead to different thermal dynamics and heat transfer conditions inside the liquid container. Thus, the FBG–air was used as the room temperature reference.

The classification results of the RF generated models are presented in the confusion matrix of Table 4, which shows an identity matrix resulting from the correct classification of the analyzed fluids. The FBG temperature sensors were able to identify the liquids in all analyzed cases. However, it is important to mention that a higher number of liquids and the temperature conditions can reduce the classification accuracy, as shown in [23], where the simulations showed that an accuracy higher than 90% can also be achieved in different fluid and heat transfer conditions. Moreover, there were intrinsic uncertainties due to the methodological and instrumental uncertainties that could influence the accuracy of the whole system. Compared with our previous work [23], the accuracy in the liquid classification increased due to the different experimental conditions of the analyses presented in this paper. In [23], the simulations were performed considering a larger storage tank and with only an estimation of the heat transfer from the Sun to the container. In contrast, the experimental analysis presented here employed a Peltier plate beneath a smaller liquid container (with 22.5 cm height). These different experimental conditions led to a larger thermal gradient along the liquid container, resulting in higher temperature differences in the liquids. As the wavelength shift was proportional to the temperature variations, this experimental condition aided inn the fluid classification using the wavelength shift of the FBGs as the input to the machine learning algorithms.

Table 5 shows the RMSE of Models 1 and 2 for each fluid for both the decreasing and increasing levels. An average RMSE of 0.2603 cm, with a maximum RMSE lower than 0.4 cm, was obtained in the liquid level measurement also using the RF algorithm. The RMSE values were thus low regarding the liquid level range from 0.9 cm to 22.5 cm. The RMSE was obtained from the liquid level estimation using regression approaches, which led to a continuous variation in the wavelength shift of each FBG as a function of the level. Thus, it was possible to obtain subcentimeter errors (and even a submillimeter resolution, as summarized in [4]), which was below the FBG physical length, as the limiting factor for the sensor liquid level estimation resolution was the wavelength resolution and accuracy of the optical interrogation. In this case, the optical interrogator used (Micron Optics, sm125) had a wavelength resolution of 1.0 pm.

**Figure 6:**
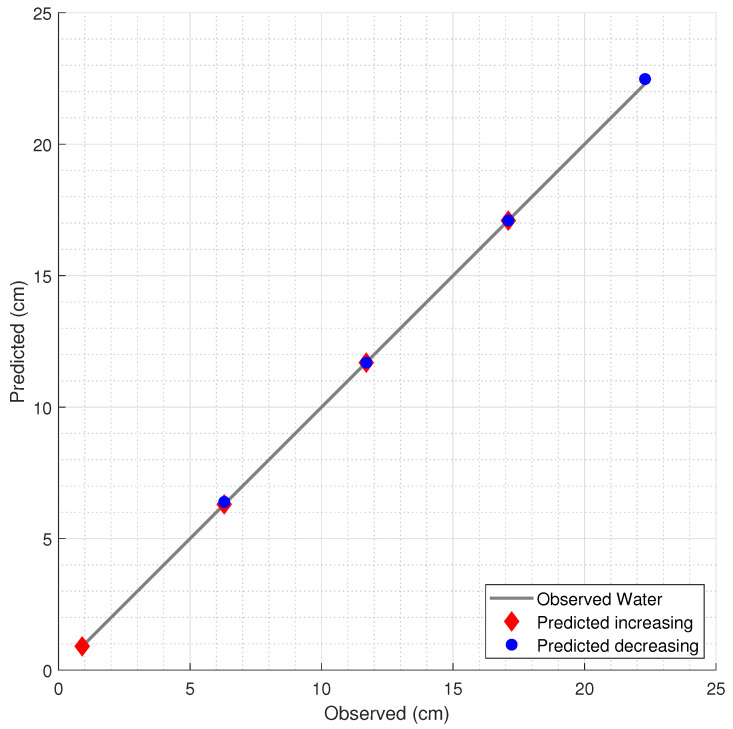
Estimation of the water level by the FBG measurements and the corresponding RF model.

**Figure 7:**
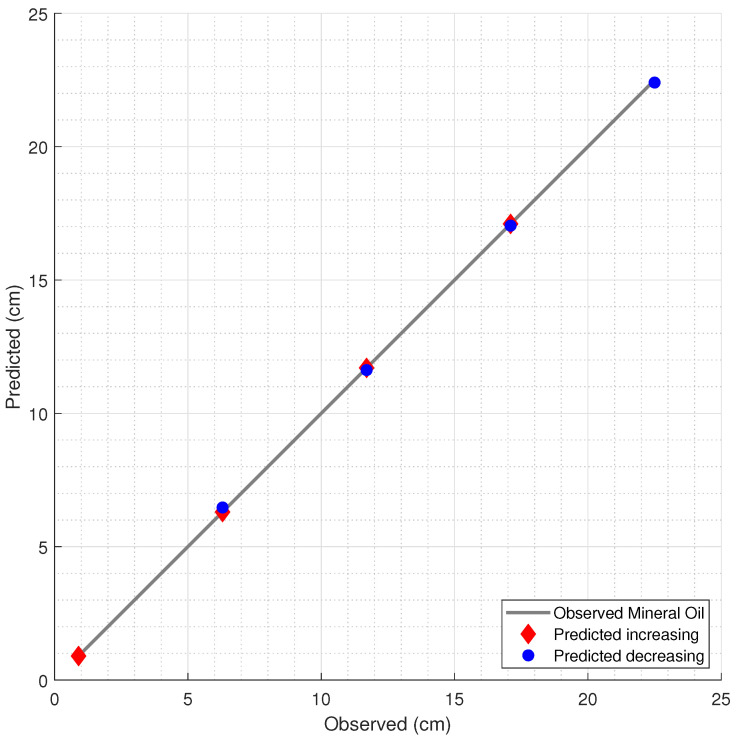
Prediction of mineral oil level variation.

**Figure 8:**
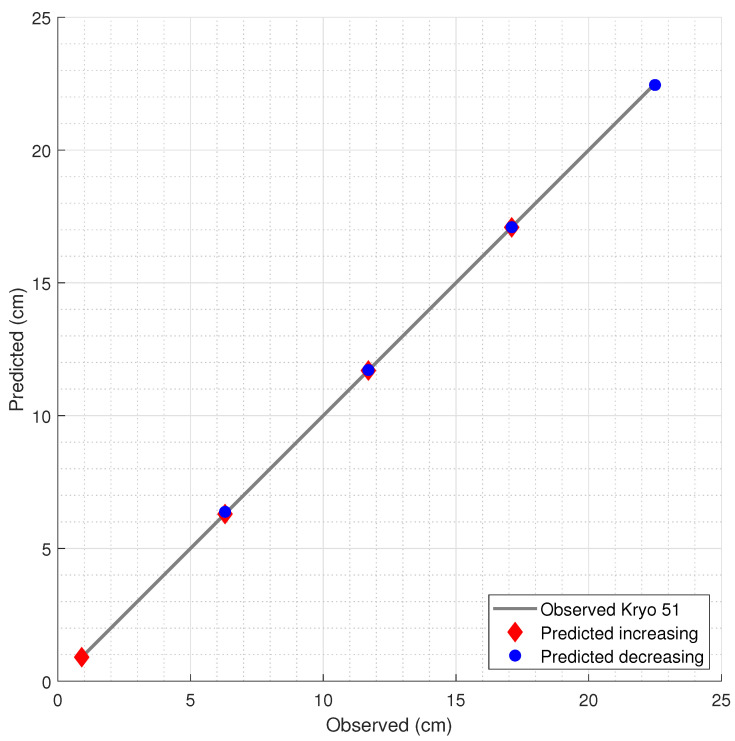
Prediction of Kryo 51 level variation.

The results of the liquid level predictions, with the estimated levels and the corresponding observed levels, are presented in Figure 6, Figure 7 and Figure 8 for water, mineral oil, and Kryo 51, respectively. Model 1 was used in the water and Kryo 51 for both the decreasing and increasing level estimations due to its lower error in regard to the corresponding error of Model 2. In the mineral oil level estimations, Model 2, which was less complex and had a lower overfitting risk, was used in both the decreasing and increasing oil predictions due to the proximity between the errors of Models 1 and 2, with an increase of 13.97% and a decrease of 6.02% in the RMSE of Model 2 in regard to that of Model 1 for decreasing and increasing levels, respectively.

## 4. Conclusions

This paper proposed the use of an array of FBG-based temperature sensors for the liquid identification and level estimation in a tank. For the analysis, the fluids were dichotomized into oil and nonoil. The algorithm inputs were Δλ and the location of the FBG from the bottom of the tank. The fluid was identified and the liquid level estimated based on the temperature variations of the liquid inside the tank. The fluid identification by the RF algorithm achieved the correct detection of the liquids in all analyzed cases. An average RMSE of 0.2603 cm, with a maximum RMSE lower than 0.4 cm, was obtained in the liquid level measurements also using the RF algorithm. The low RMSE of the estimations, regarding the liquid level range from 0.9 cm to 22.5 cm, along with its high accuracy in classification, indicated the feasibility of using RF algorithms in fluid identification and liquid level estimations. Thus, this work indicated a novel approach for liquid level estimation in conjunction with liquid classification using a single sensor array, which provides economical and operational advantages in practical applications. The dichotomization into oil and nonoil can result in the separation between the oil (used in the next steps of its processing) and the other liquids (or liquid mixtures) generated in oil extraction and manipulation such as water and stable emulsions, even when these data are not known by the operators. Future works include the liquid classification and liquid level estimations in multiphase liquids and oil–water mixtures, as well as in different fluids and temperature variation conditions.

## Figures and Tables

**Table 1:** Pearson correlation coefficients among the observed FBGs for the decreasing and increasing water level measurements.

Water	Decreasing Level			
	FBG 1	FBG 2	FBG 3	FBG–Air
FBG 1	1.00	−0.53	−0.13	0.6
FBG 2		1.00	0.01	−0.13
FBG 3			1.00	−0.02
FBG–Air				1.00
	**Increasing Level**			
	**FBG 1**	**FBG 2**	**FBG 3**	**FBG–Air**
FBG 1	1.00	−0.28	−0.17	0.60
FBG 2		1.00	0.04	−0.18
FBG 3			1.00	−0.13
FBG–Air				1.00

**Table 2:** Pearson correlation coefficients among the observed FBGs for the decreasing and increasing mineral oil level measurements.

Mineral Oil	Decreasing Level			
	FBG 1	FBG 2	FBG 3	FBG–Air
FBG 1	1.00	−0.02	0.11	0.31
FBG 2		1.00	0.69	0.53
FBG 3			1.00	0.77
FBG–Air				1.00
	**Increasing Level**			
	**FBG 1**	**FBG 2**	**FBG 3**	**FBG–Air**
FBG 1	1.00	0.08	0.42	0.77
FBG 2		1.00	0.82	0.62
FBG 3			1.00	0.86
FBG–Air				1.00

**Table 3:** Pearson correlation coefficients among the observed FBGs for the decreasing and increasing Kryo 51 level measurements.

Kryo 51	Decreasing Level			
	FBG 1	FBG 2	FBG 3	FBG–Air
FBG 1	1.00	−0.36	0.60	0.48
FBG 2		1.00	0.03	−0.17
FBG 3			1.00	0.15
FBG–Air				1.00
	**Increasing Level**			
	**FBG 1**	**FBG 2**	**FBG 3**	**FBG–Air**
FBG 1	1.00	−0.25	−0.61	0.53
FBG 2		1.00	−0.34	0.09
FBG 3			1.00	0.03
FBG–Air				1.00

**Table 4:** Confusion matrix for fluid identification.

	Water	Mineral Oil	Kryo 51
Water	1	0	0
Mineral Oil	0	1	0
Kryo 51	0	0	1

**Table 5:** RMSE, in cm, of the proposed models for the decreasing and increasing liquid level measurements of each fluid.

Fluid	RMSE (Decreasing Level)		RMSE (Increasing Level)	
	Model 1	Model 2	Model 1	Model 2
Water	**0.367**	1.620	**0.382**	1.712
Mineral Oil	0.136	**0.155**	0.166	**0.156**
Kryo 51	**0.247**	0.446	**0.255**	0.444

## Data Availability

Data available by reasonable request.
